# Dynamic graph convolutional network for assembly behavior recognition based on attention mechanism and multi-scale feature fusion

**DOI:** 10.1038/s41598-022-11206-8

**Published:** 2022-05-05

**Authors:** Chengjun Chen, Xicong Zhao, Jinlei Wang, Dongnian Li, Yuanlin Guan, Jun Hong

**Affiliations:** 1 School of Mechanical and Automotive Engineering, Qingdao University of Technology, Qingdao, 266520 Shandong China; 2 School of Mechanical Engineering, Xi’an Jiaotong University, Xi’an, 710049 Shanxi China

**Keywords:** Mechanical engineering, Electrical and electronic engineering

## Abstract

Intelligent recognition of assembly behaviors of workshop production personnel is crucial to improve production assembly efficiency and ensure production safety. This paper proposes a graph convolutional network model for assembly behavior recognition based on attention mechanism and multi-scale feature fusion. The proposed model learns the potential relationship between assembly actions and assembly tools for recognizing assembly behaviors. Meanwhile, the introduction of an attention mechanism helps the network to focus on the key information in assembly behavior images. Besides, the multi-scale feature fusion module is introduced to enable the network to better extract image features at different scales. This paper constructs a data set containing 15 types of workshop production behaviors, and the proposed assembly behavior recognition model is tested on this data set. The experimental results show that the proposed model achieves good recognition results, with an average assembly recognition accuracy of 93.1%.

## Introduction

Currently, manual assembly is still an important assembly form of discrete production. Personnel is one of the key elements affecting product assembly quality and efficiency. Especially for assembly workshops with a large number of personnels, irregular assembly behaviors will greatly reduce the production efficiency, quality, and safety of the workshop. Therefore, assembly behavior monitoring is crucial for lean management and intelligent production in discrete manufacturing enterprises. Traditional personnel monitoring methods usually collect real-time images of the workshop and send them to the monitoring center, where the monitoring team supervises the production and assembly process of the workshop personnel. Due to the large number of production stations and production staff in the workshop, it is difficult for the monitoring team to monitor the entire workshop both in real -time and globally, especially for the assembly workshop with a large area. To monitor the entire workshop and prevent irregularities, such as smoking and violent handling, it is necessary to install multiple cameras in every corner of the workshop, and a large number of people are required to monitor the workshop through these cameras. However, it is difficult for the monitoring personnel to watch the video of assembly behavior in the workshop for a long time, which can cause missing monitoring. Hence, traditional manual video surveillance methods are not suitable for workshop monitoring with largescale, multiple equipment, and complex environment.

Nowadays, the behavior monitoring of workshop personnel is developing in the direction of digitization and intelligentization. Intelligent behavior recognition and monitoring methods are mainly divided into two categories: methods based on human motion signals^1,2^ and methods based on vision^3–7^. The former mainly collects the position, motion, or surface EMG signals of human joints by the sensors worn by workers and recognizes behaviors by analyzing these signals. This kind of method requires the workers to wear special sensors, which can affect production. The latter recognizes behaviors by analyzing video images, and the workers do not need to wear sensors. This kind of method has gradually attracted the attention of researchers from all over the world. The early research mainly extracts image features (optical flow features and human skeleton) based on prior knowledge and then performs human action recognition^8–10^ through traditional machine learning methods, such as hidden Markov model (HMM) and support vector machine (SVM). The current methods based on artificial feature extraction can no longer meet the requirements of high precision and high speed. The application of deep learning models can automatically extract assembly action features, avoiding the complexity and difference of manual feature extraction. Thus, assembly behavior recognition based on deep learning is an important development trend.

Aiming at the intelligent monitoring of assembly behavior based on deep learning, this paper proposes a dynamic graph convolutional network model for assembly behavior recognition based on attention mechanism and multi-scale feature fusion. The multi-scale feature fusion module extracts features of different scales through dilated convolution with different ratios to avoid losing global information by the deep network. The attention mechanism superimposes the attention weights of different feature channels and feature spaces with image feature information. This makes the network model focus on the key information in the image and enhances the recognition ability of the model to a certain extent. The dynamic graph convolution can dynamically capture the potential relationship between human actions and assembly tools in assembly behavior images, further enhancing the model’s characterization ability.

This paper is organized as follows. Section "Related works" summarizes the related research work for assembly behavior recognition. Section “Methods” describes the dynamic graph convolutional network model for assembly behavior recognition based on attention mechanism and multi-scale feature fusion. In section "Data set establishment", the assembly behavior recognition data set is established. In section "Experiments", ablation experiments and comparative experiments are conducted to prove the validity of the method proposed in this study. Section "Conclusion and future work" presents the conclusions and future work.

## Related works

In the field of assembly behavior recognition, the existing methods can be roughly divided into two categories according to the type of data acquired by sensors: wearable sensor-based motion recognition and visual sensor-based motion recognition. Common wearable sensors include acceleration sensors, gyroscope sensors, and magnetometers. These sensors can be worn directly or integrated into portable devices, such as MYO armbands, arm bands, and data gloves. When attached to the human body, these sensors can obtain human body motion data such as acceleration signals, azimuth signals, and electromyographic signals. Then, the obtained data is processed with machine learning models such as Hidden Markov Model (HMM)^11^, Support Vector Machine (SVM)^12^, and K-Nearest Neighbors (KNN)^13^ for action recognition. Kutafina et al.^14^ collected EMG signals of six hand-washing actions through an MYO armband and used HMM to recognize the hand-washing actions. This method achieved a recognition accuracy of 98.3%, indicating a good recognition effect. Stiefmeire et al.^15^ used ultrasound and motion sensors to collect weak signals of human hand positions and used HMM to identify the hand movements of workers in bicycle maintenance scenarios. Jiang et al.^16^ proposed a method for segmentation and recognition of human body motions in the mechanical assembly process based on SVM. Koskimaki et al.^17^ used wrist-worn IMU sensors to capture arm movements and used the KNN model to classify the five assembly activities on the industrial assembly line.

Due to its insufficient flexibility, low scalability, and poor user experience, the action recognition method based on wearable sensors can only be used in specific scenarios. By contrast, the action recognition method based on visual sensors does not have these limitations, and it has been widely studied and used. The process of this type of method is as follows: first, features are extracted manually; then, a model representing human actions is built; finally, action recognition is performed based on the established model. Among the traditional vision-based methods, the action recognition method of DT (Dense Trajectories)^18^ and iDT (improved Dense Trajectories)^19^ achieves the best recognition effect. DT first intensively samples the feature points of the images in the video; then, it tracks the feature points to obtain the trajectories in the video sequence and perform feature extraction and coding based on these trajectories; finally, it performs actions by using machine learning methods such as SVM classification. iDT matches the optical flow between the two frames before and after the video and the SURF key points to reduce the impact of camera movement and make the features more concentrated for the description of human movement. Warren et al.^9^ used the optical flow information in the image to control people's walking. Somayeh et al.^10^ proposed an action recognition algorithm that uses optical flow to construct motion descriptors and uses SVM for classification. This method can recognize the action of walking, running, jogging, clapping, waving, and boxing. Also, it can be applied to the video with a low framerate without reducing the recognition accuracy, and it has a certain degree of robustness under different viewing angles. Although the method based on optical flow performs well on motion recognition, it cannot meet the real-time requirement of assembly motion tasks because the algorithm has a low calculation speed.

The traditional method of constructing complex models based on manual extraction of action features fails to achieve high speed and high precision. With the improvement of computing power and the proposal of more powerful models, the action recognition method based on deep learning has become popular. The deep learning model performs deep feature extraction and action classification through an end-to-end neural network structure, which can avoid the difference in manual feature extraction^20^. In recent years, more and more deep learning networks have been applied to action recognition. Especially, Convolutional Neural Network (CNN)^21^ has unique advantages in image processing because of its special structure and layout of local weight sharing that are closer to the actual biological neural network. Graph Convolutional Network (GCN)^22^ is also widely used because of its superiority in modeling non-Euclidean space structures.

The current deep learning-based action recognition method consists of two parts: 1) an image recognition method to extract and classify spatial–temporal features; 2) a pose estimation method to extract skeleton information for retraining. The representative action recognition network models mainly include two-stream CNN, 3D CNN, and GCN. The basic principle of the two-stream CNN model is to calculate the optical flow difference for every two frames in the image sequence to obtain the dense optical flow. Then the CNN model is trained on the action image and the dense optical flow separately, and the action category is judged. Finally, the training results of the two networks are fused to obtain the final classification result. The dual-stream network proposed by Simonyan et al.^23^ uses a two-branch network architecture. The network uses RGB images as input to extract the spatial -domain features and uses optical flow information as an input to extract the time -domain features, and it uses a multi-task training method. The classifications performed on two behavior recognition data sets of UCF-101 and HMDB-51 show that the network achieved good recognition results. Feichtenhofer et al.^24^ improved the dual-stream network model by integrating a spatial network and a time -series network, and five different integration solutions were proposed. This improvement promoted the development of the dual-stream network architecture.

The method of applying convolutional neural network in action recognition is to use CNN to recognize each frame of image, but this method ignores the motion information between consecutive frames and can only capture the spatial information within the frame. To effectively integrate motion information, many researchers have begun to use the 3D CNN method. Tran et al.^25^ proposed a simple and effective C3D network model that uses 3 × 3 × 3 three-dimensional convolution kernels to learn spatial–temporal features of action images. Previous 3D CNN fails to main fully mine the long-term time -domain information, and there are excessive network parameters. To overcome these drawbacks, Dibaet al.^26^ proposed an end-to-end network model called T3D (Temporal 3D-ConvNets). The time -domain 3D convolution kernel and TTL (Temporal Transition Layer) can effectively capture high-level semantic information in the long-term temporal domain. Since this 3D CNN model extracts the temporal and spatial characteristics of the image through the 3D convolution kernel, it can capture more abundant motion information. Also, it has a high operation speed. These features make 3D CNN have a good application prospect, but its recognition accuracy is generally lower than the two-stream CNN network.

In recent years, more and more researchers have realized that action recognition tasks require higher-dimensional features. One of the solutions is to introduce graph convolutional networks to discover and express action features because graph convolution^22^ has better learning ability when dealing with topological data. Yan et al.^27^ proposed a spatial–temporal graph convolutional network for model bone modeling, and it is the first graph convolutional network for behavior recognition tasks. First, the spatial features of the skeleton are extracted through graph convolution; then, the temporal features are obtained through temporal convolution; finally, the temporal features and spatial features are fused to obtain the action classification results. Shi et al.^28^ proposed a dual-stream-based adaptive graph convolutional network. By using the backpropagation algorithm, the topology map can be learned adaptively based on the joint point information, thereby increasing the flexibility of the model. The dual-stream model takes the joint point information of the human skeleton and the length and direction information of the bones as the network input to significantly improve the accuracy of the model. However, only relying on human bone nodes for action recognition leads to some problems. For example, the assembly behavior in the workshop is often related to the use of tools. The bone nodes can only express the behavior of the human body, so the assembly action is often ignored. Graph convolutional networks are not only used to recognize human actions but also have good effects in modeling the relationship between categories in image objects. Chen et al.^29^ proposed a model based on static image convolution to model the relationship between categories to improve image recognition capabilities. Ye et al.^30^ proposed to use dynamic graph convolution to dynamically generate a specific graph structure for each image, which improved the generalization ability of the network model to a certain extent. Attention mechanism can help the graph convolutional networks to focus on the key information. Liu et al.^31^ propose an enhanced attention-tracking method, combined with multi-branch network, for egocentric activity recognition. A class-aware attention maps was proposed to employ a self-attention-based module to refine the class activation maps. Liu et al.^32^ also propose a multimodal-semantic context-aware graph neural network to capture rich visual and semantic contexts.

It can be seen from the above research that deep learning-based human action recognition has been extensively studied, but the existing methods often only recognize human actions. For industrial assembly action recognition, these methods often cannot accurately determine the type of assembly behavior only by judging human actions. Therefore, it is also crucial to recognize the operation object. In this paper, the graph convolutional network model is used to establish the potential relationship between human actions and operating tools or objects, so as to recognize assembly behaviors and improves the recognition ability of the model to a certain extent. Meanwhile, in the industrial production environment, many operating tools are small in size. The existing action recognition network models often have insufficient recognition accuracy for such objects. In this paper, a multi-scale feature fusion module is introduced into the graph convolutional network model, and the high-resolution low-level feature information in the feature map is fused with the semantic information of the high-level feature, which greatly improves the model's recognition accuracy for small operating tools.

## Methods

**Figure 1:**
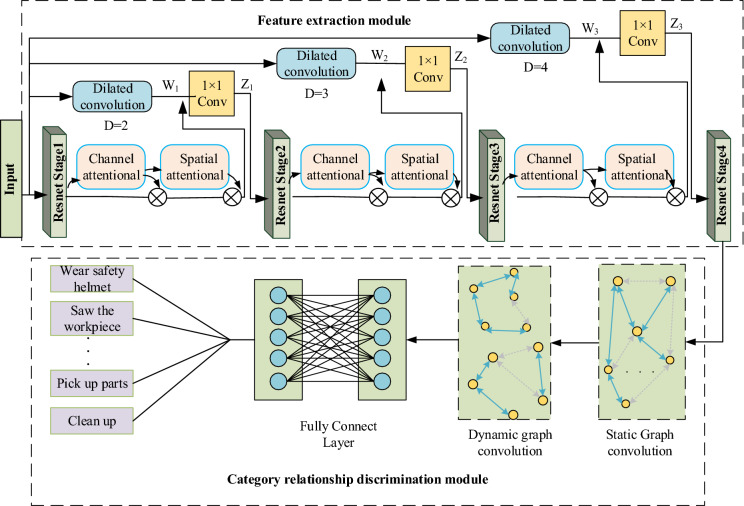
Overall network architecture.

This paper proposes a dynamic graph convolutional network model called AM-GCN for assembly action recognition based on attention mechanism and multi-scale feature fusion. Figure 1 shows the overall architecture of the network. The network is mainly composed of two parts: the image feature extraction module and the category relationship discrimination module. The image feature extraction module takes the residual network Resnet101^33^ as the backbone network for network feature extraction, which mainly consists of four residual network layers (stage1, stage2, stage3, and stage4). Meanwhile, an attention mechanism and a multi-scale feature fusion method are proposed. A spatial attention module and a channel attention module are added between each residual layer. Features are extracted in the channel and spatial dimensions, and attention is weighted. The weighted features are used as the input of the next residual layer. Besides, to reduce the information loss caused by the excessively deep network layers and to enhance the ability of the network to extract multi-scale image features, this paper uses dilated convolutions with different expansion ratios to obtain feature information of different scales. Also, the different expansion ratios of convolutions make the extracted local receptive fields different. The features extracted by the first three residual layers introduced into the attention mechanism are then fused with the feature information obtained through dilation convolutions, thereby extracting action features in images and target tool features of different scales.

The category relationship discrimination module uses the human action features and assembly tool features extracted in the feature extraction stage as the input of static and dynamic graph convolutions. The static graph convolution adopts the stochastic gradient descent method to initialize the adjacency matrix. Since the adjacency matrix shares parameters for all training images, the global category dependencies can be captured. However, in dynamic graph convolution, the adjacency matrix is different for each image, which improves the model’s expression ability and reduces the risk of overfitting caused by the static graph convolution. Through the joint training of static graph convolution and dynamic graph convolution, the self-adaptive capture of the potential relationship between different assembly actions and assembly tools in the image is achieved, thereby realizing assembly behavior recognition.

### Attention module

The recognition of assembly behavior in the workshop is to recognize the actions of workshop personnel, production operation tools, and the relationship between them. However, operating tools usually occupy a small proportion of the image and are easily affected by external environmental factors, such as the items in the workshop (workbenches and machine tools). In this case, the network model fails to accurately capture the feature information of the assembly tools in the image, thus affecting the recognition of assembly behaviors. Besides, when the image is processed by multi-layer convolution operation, the feature matrix of multiple channels can be obtained. However, not all channels have important information. Therefore, it is necessary to filter useless channels to perform feature optimization. In response to the above problems, this paper adds a spatial attention module and a channel attention module to the network model^34^. Also, different weight information is assigned to different feature dimensions and feature channels of the image to measure the importance of each channel. The spatial attention mechanism enables the network model to pay more attention to the spatial position information on the feature map. The spatial attention module and the channel attention module are embedded between the adjacent residual networks to obtain more useful information in the channel and space dimensions for assembly behavior recognition, thereby improving the recognition ability of the network.

**Figure 2:**
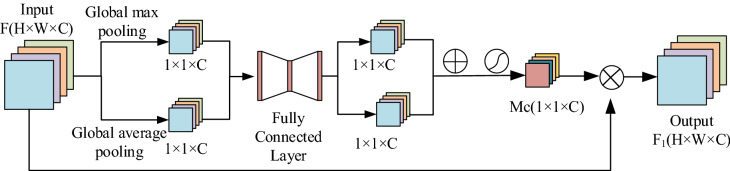
Channel attention module.

The channel attention module can enhance the weight of the useful information and suppress the weight of the useless information in the feature channel. In this way, the network model focuses more on the discriminative channels in the image. The structure of the channel attention module is shown in Fig. 2. Firstly, the global maximum pooling and global average pooling operations are performed on the feature ${\text{F}}$ whose input dimension is ${\text{W}} \times {\text{H}} \times {\text{C}}$ to obtain two channel descriptions with a dimension of $1 \times 1 \times {\text{C}}$. The application of two different pooling operations helps to obtain richer high-level features. Then, the obtained channel descriptions are sent to a two-layer convolutional neural network that models the correlation between channels. Finally, the two output features are added, and the new attention weight coefficient $M_{c}$ is obtained through the sigmoid activation function. The input feature F is multiplied by the weight coefficient $M_{c}$ to obtain the re-calibrated feature $F1$ in the channel dimension, as shown in formula (1):

1 $$ F1 = M_{c} \left( F \right) = \sigma \left( {Conv\left( {AvgPool\left( F \right)} \right) + Conv\left( {MaxPool\left( F \right)} \right)} \right) $$

where $\sigma$ represents sigmoid activation function, and $Conv()$ represents a two-layer convolutional neural network.

**Figure 3:**
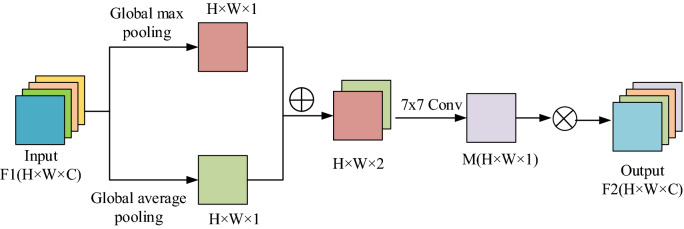
Spatial attention module.

The network structure of the spatial attention module is shown in Fig. 3. This module performs feature filtering on the pixels at different positions in the same channel in the spatial dimension, and it weights the features of useful positions, thereby enhancing the spatial position information in the feature map. This module takes the attention feature $F_{1}$ generated by the channel attention network as the input feature. First, the maximum pooling and average pooling are performed respectively to obtain two feature maps with a channel number of 1. The two feature maps are spliced in the channel dimension. Then, a 7 × 7 convolution with a softmax activation function is performed for feature learning. Finally, the final spatial position weight coefficient $M$ is obtained. The feature $F_{1}$ is multiplied by the weight coefficient $M$ to obtain a new attention feature $F_{2}$, as shown in formula (2):

2 $$ F2 = M\left( {F_{1} } \right) = \sigma \left( {f^{7 \times 7} \left( {\left[ {AvgPool\left( {F_{1} } \right),MaxPool\left( {F_{1} } \right)} \right]} \right)} \right) $$

### Multi-scale feature fusion module

Because the size of different tools in the assembly behavior image is inconsistent, this has a large impact on the recognition of assembly behaviors. Meanwhile, if the convolutional neural network uses convolution kernels of the same size to extract the features of the assembly behavior image, the receptive field of the network model is limited. In this case, only the local information in the image can be extracted, and the rich semantic information will be lost, which is not conducive to recognizing specific information in complex scenes. To increase the receptive field of the model without increasing the calculation amount, the spatial resolution of the image can be reduced by downsampling operations such as pooling. Besides, to solve the problem of reduced recognition accuracy caused by the inconsistent size and scale of the assembly tools in the assembly behavior image, this paper designs a multi-scale feature fusion method, which can recognize more accurate feature information between specific assembly behaviors and environments.

As shown in Fig. 1, firstly, a convolution operation with a kernel size of 7 × 7 and a maximum pooling operation is performed on the input image to obtain a feature $M$ with a dimension of 64 × 112 × 112. Then, the obtained feature map $M$ is input into the dilated convolutions with three expansion ratios and filling sizes of 2, 3, and 4, and step sizes of 2, 4, and 8 to obtain three feature maps with scale sizes of $W{}_{1},W_{2} ,W{}_{3}$. Subsequently, $W{}_{1},W_{2} ,W{}_{3}$ are respectively fused with the output features of the residual modules Stage1, Stage2, and Stage3, to obtain new features $Z_{1} ,Z_{2} ,Z_{3}$ with multi-scale information. Finally, convolution with a kernel size of 1 × 1 is performed to reduce the dimensionality of $Z_{1} ,Z_{2} ,Z_{3}$ in the channel dimension, and there output is respectively input into the residual module of the next stage to extract multi-scale information.

### Graph convolution module

Traditional convolutional neural networks have achieved great success in processing images with regular spatial data. However, they cannot effectively process irregular non-Euclidean space data, such as learning the potential relationship between human pose and assembly tools in assembly behavior images. In the assembly action recognition task, it may not be enough to rely only on the differences in human actions to identify different assembly actions. For example, in the actions of smoking and drinking, the movements of the human body are similar, that is, the arms are raised and bent. However, if it is easier to identify the difference between the two categories using tools associated with human actions (cigarettes and water glasses), the effect of action recognition can be further improved. The original ADD-GCN builds a dynamic graph that can get the category relationships of perceptible content in images, such as the latent relationship between human actions and assembly tools, which further enhances the model’s representational capabilities.

Graph-based learning methods have become increasingly popular in capturing complex relationships between objects. Graphs can be a common method to express label dependencies. For graph ${\text{G}} = \left( {V,E} \right)$, *V* represents the set of nodes in the graph, and E represents the set of edges connecting the nodes. Each node i has its own features $X_{i}$. These features can be represented by a matrix $X_{N*D}$, where N is the number of nodes, and D is the feature dimensionality. Besides, each node in the graph has structural features, that is, there is a certain connection between a node and another. Generally, both node features and structural features (i.e., features of the edges) should be considered for the graph data.

Graph convolutional neural network is a deep learning method for processing graph data. It can automatically learn node features and the associated information between nodes. Taking human action features and tool features as the node information of a graph, this paper adopts graph convolutional networks to mine the potential relationships between the two types of features to improve the recognition accuracy of the network model.

Currently, it is a common method to exploit the co-occurrence frequency between labels to establish a complete graph structure and learn the relationship between categories through graph convolutional neural networks. For example, based on the co-occurrence relationship between labels, Chen et al.^29^ employed static graph convolution to guide the image feature extraction network to perform multi-label recognition. However, this method constructs the correlation matrix A in a static manner, so the content in each input image is not sufficiently used. To solve this problem, Ye et al.^30^ proposed a dynamic graph convolution method to dynamically generate a specific graph structure for each image. This method does not need to set a static adjacency matrix in advance. It automatically generates an adjacency matrix for a specific image. To some extent, the over-reliance on the label co-occurrence relationship is avoided, and the generalization ability of the network model is improved. Based on the network model proposed by Ye et al.^30^, this paper uses the joint training method of static and dynamic graph convolutional networks to model the potential relationship between tools and actions in the assembly behavior image. Also, a dynamic graph convolutional network architecture suitable for assembly behavior recognition is designed.

**Figure 4:**
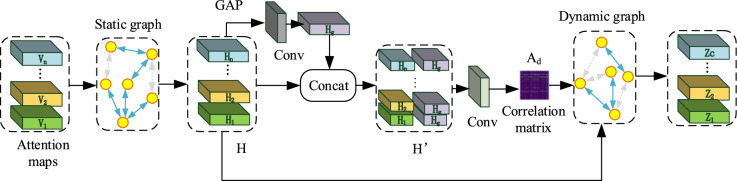
Dynamic graph convolution module.

In this paper, the structure of the graph convolutional network proposed by Ye et al.^30^ is shown in Fig. 4. It consists of two parts: a static graph and a dynamic graph. The model uses the feature output $F_{2}$ of the feature extraction module as node feature $V$, and $V$ is input to static graph convolution and dynamic graph convolution. Firstly, the static graph convolution adopts the stochastic gradient descent method to initialize the adjacency matrix A and the static graph weight matrix W. Then, the updated node feature H is obtained, which can be expressed as $H = ReLU\left( {AVW} \right)$, where $H = \left[ {H_{1} ,H_{2} , \ldots ,H_{n} } \right] \in R^{{C \times D_{1} }}$. Because the static adjacency matrix A is parameter-sharing on all images, the global category dependencies can be captured to a certain extent. Then, the global average pooling, regularization, and one-layer convolution operation are successively performed on H to obtain the global feature representation $H_{g} \in R^{{D_{1} }}$. Subsequently, $H$ and $H_{g}$ are connected through a jump connection to obtain $H^{\prime}$. Different from the static graph convolution, the adjacency matrix $A_{d}$ of the dynamic graph is adaptively estimated through $H^{\prime}$. The adjacency matrix of the static graph is fixed and is shared for all input images during the training process. The adjacency matrix A of the dynamic graph changes dynamically according to the input image. Different inputs images have different $A_{d}$, improving the expression ability of the model and reducing the risk of overfitting caused by static graphs to a certain extent. Finally, the final output Z is obtained through joint training of static graph convolution and dynamic graph convolution, which is expressed in formula (3)

3 $$ Z = f\left( {A_{d} HW_{d} } \right) $$

where $f\left( . \right)$ represents the LeakyReLU activation function, and $W_{d}$ represents the weight matrix.

## Data set establishment

**Figure 5:**
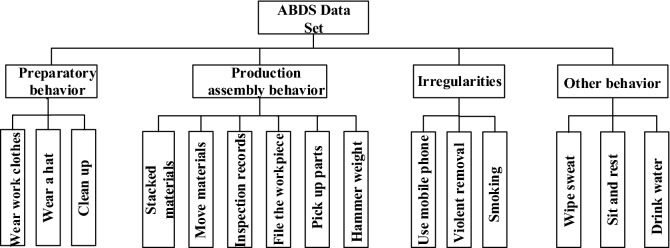
Action overview in ABDS data set.

To verify the validity of the model proposed for assembly behavior recognition, simulation and data collection of production assembly behaviors are performed. Then, the assembly behavior data set (ABDS) is generated. The production behaviors of workshop personnel are divided into four categories: preparatory behaviors, production and assembly behaviors, irregular behaviors, and rest behaviors (Fig. 5).

1. Preparatory behaviors are the preparatory work that needs to be completed before workshop personnels enter the manufacturing workshop. In some workshops with strict production environment requirements, the preparatory work is particularly important. Hence, three actions (wearing work clothes, wearing work hats, and cleaning) are selected as preparatory behaviors.
2. The assembly process in the workshop involves the handling of materials. During the production process, it may involve the processing of workpiece, including filing the workpiece and hammering heavy objects. Also, picking up parts is common. The final inspection is usually conducted after the assembly is completed. Hence, the inspection record is included.
3. Irregularities can have a certain impact on the production process, so timely detection of such behaviors helps to improve production safety. Common irregularities include irregular use of communication equipment, smoking, and violent handling.
4. Other behaviors, such as drinking water, having a rest, and wiping sweat, are common in the workshop and do not violate the regulations. However, sometimes they affect production efficiency.

**Figure 6:**
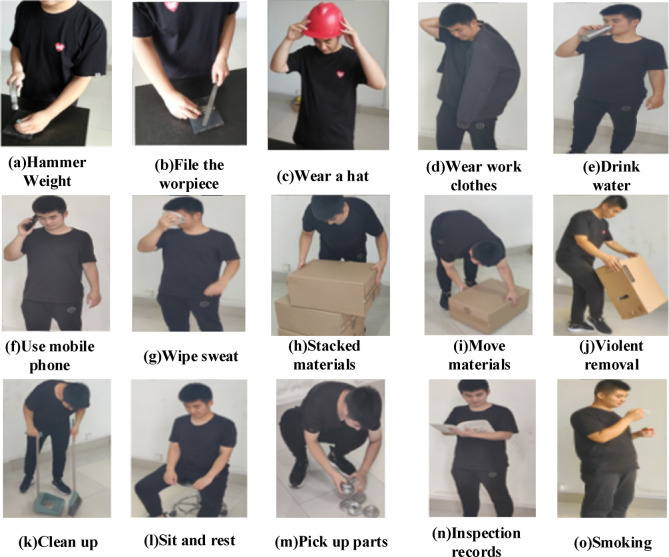
Data set samples.

To build the ABDS, this experiment uses an RGB-D camera to capture the behaviors. Six people with an average age of around 25 participated in the shooting of the images of different behaviors. Each person completed all the behaviors, and each behavior was repeated 3 times. Each behavior contains 320 images, so the dataset contains a total of 4800 samples. 80% of the entire dataset is randomly selected as the training set, 10% as the validation set, and the other 10% as the test set. Some examples of the behaviors in the ABDS data set are shown in Fig. 6.

## Experiments

This section firstly introduces the experimental parameter settings and evaluation indexes of the network model. Then, the effectiveness of the proposed network model for assembly behavior recognition is verified on the ABDS data set. This section also analyzes the experimental results.

### Experimental parameter settings

The experiment is conducted on a computer equipped with Intel Xeon E5-2630 CPU processor, and four NVIDIA TITAN Xp graphics cards. The computer runs Ubuntu 18.04 operating system. The network training and testing are performed using the PyTorch 1.10 framework. 80% of the data set is randomly selected as the training set, and 20% as the test set. A data enhancement method is applied to reduce the risk of overfitting. The input image is randomly cropped to have a size of 448 × 448 and randomly horizontally flipped. Besides, the ReLU activation function is used. The batch size is set to 30, and a total of 200 epochs are trained. The learning rate of the first 30 epochs remains unchanged. Then, the learning rate is attenuated by 10% for every 30 epochs. SGD is used as the optimizer. Additionally, the momentum is set to 0.9, with a weight attenuation coefficient of 10^–4^.

### Evaluation indexes

Currently, precision rate, recall rate, and F metric (F1) are common indexes to evaluate the performance of a model. The precision rate is the probability that the prediction is correct. The recall rate is the probability that the positive sample is predicted to be correct. The F metric is the harmonic average of the precision rate and the recall rate. Since the F metric comprehensively considers recall rate and precision rate, it has a relatively large impact on performance evaluation. In the experiment, according to the indexes used by previous studies^35^, overall recall rate (*OR*), overall precision (*OP*), overall F1 value (*OF1*), category precision (*CP*), category recall rate (*CR*), and category F1 value (*CF1*) are selected as evaluation indexes. Their calculation of these indexes is shown in formula (4).

4 $$ \begin{aligned} & OP = \frac{{\mathop \sum \nolimits_{i} N_{i}^{c} }}{{\mathop \sum \nolimits_{i} N_{i}^{P} }}, \;\;CP = \frac{1}{C}\mathop \sum \limits_{i} \frac{{N_{i}^{c} }}{{N_{i}^{P} }} \\ & OR = \frac{{\mathop \sum \nolimits_{i} N_{i}^{c} }}{{\mathop \sum \nolimits_{i} N_{i}^{g} }},\;\;CR = \frac{1}{C}\mathop \sum \limits_{i} \frac{{N_{i}^{c} }}{{N_{i}^{g} }}{ } \\ & OF1 = \frac{2 \times OP \times OR}{{OP + OR}}, \;\;CF1 = \frac{2 \times CP \times CR}{{CP + CR}} \\ \end{aligned} $$

where *C* represents the number of labels; $N_{i}^{c}$ is the number of images that are correctly predicted for the i-th label; $N_{i}^{P}$ is the number of images that are predicted to be the i-th label; and $N_{i}^{g}$ is the actual number of images for the i-th label.

### Ablation experiments

**Figure 7:**
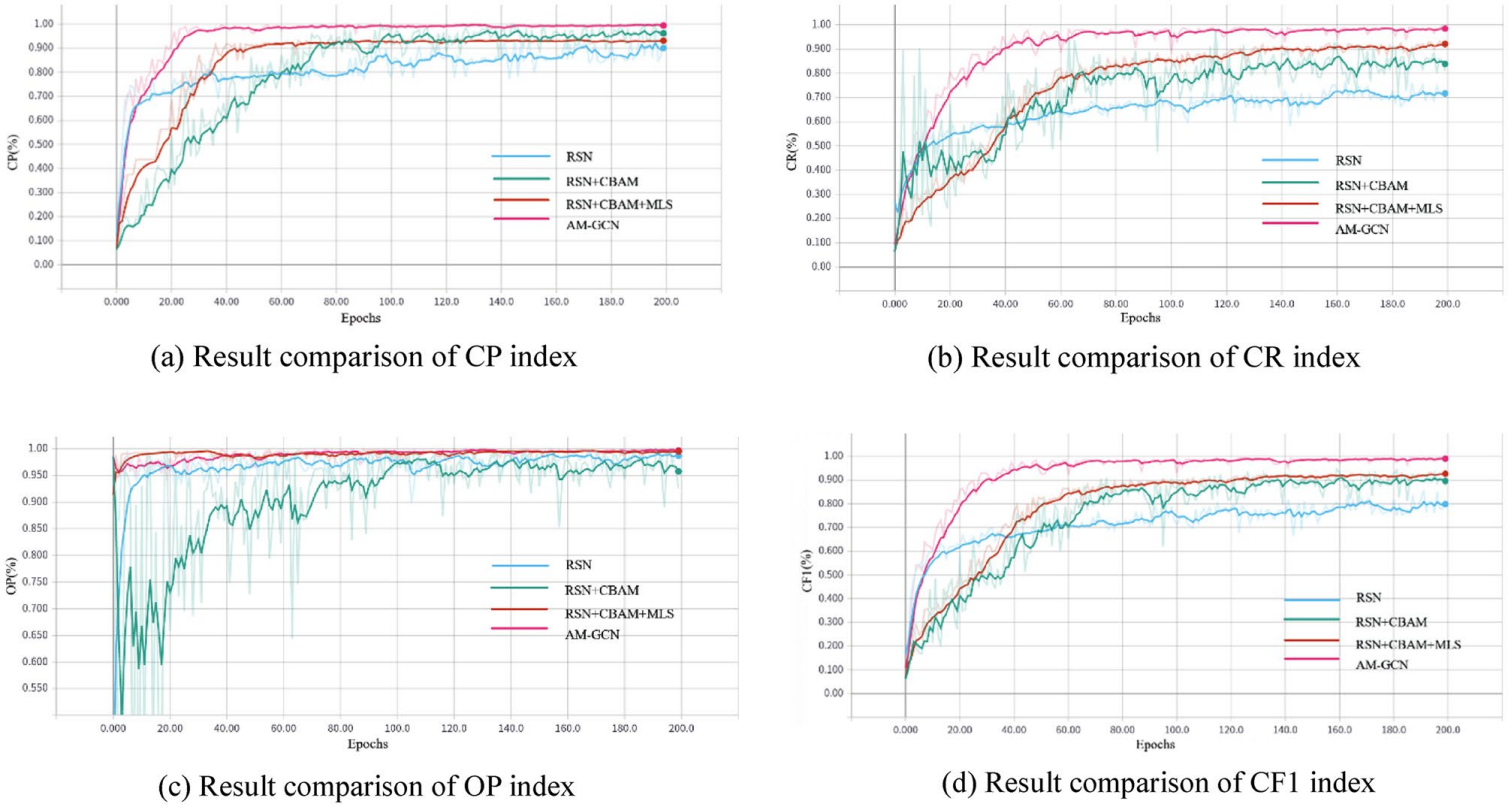
Comparison results of different indexes.

To study the contribution of each module in the proposed network model, ablation experiments of the modules are performed on the ABDSdata set. The original Resnet101 framework (abbreviated as RSN) is taken as the baseline. RSN + CBAM refers to adding a channel spatial attention module to Resnet101; RSN + CBAM + MLS refers to adding a multi-scale fusion module to RSN + CBAM; AM-GCN is the network model proposed in this paper. Figure 7 shows the training results of these four experiments in terms of CR, CP, OP, and CF1. The Resnet101 network performs the worst in CR, CP, and CF1. Adding channels and spatial attention to Resnet101 can improve network performance to varying degrees, and the performance increases by about 8% in CR and CF1. However, adding a multi-scale feature fusion module to Resnet101 with the attention mechanism improves the performance by about 2% in CR. The network model proposed in this paper performs the best in CR, CP, OP, and CF1. The experimental results indicate that the added modules help to improve the performance of the model.

### Comparative experiments

**Figure 8:**
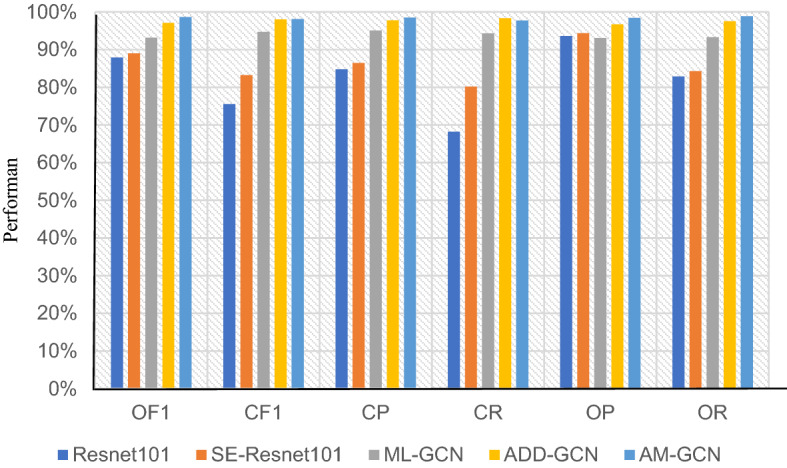
Comparison results of different classification models.

To verify the effectiveness of the proposed AM-GCN model, deep learning-based classification models including Resnet101^33^ (deep residual learning for image recognition), ML-GCN^29^ (multi-label image recognition with graph convolutional networks, and ADD-GCN^30^ (attention-driven dynamic graph convolutional network for multi-label image recognition), SE-Resnet101^36^ (squeeze-and-excitation networks) are taken for performance comparison. The comparison results of each index are shown in Fig. 8.

As shown in Fig. 8, the proposed AM-GCN achieves good performance under different evaluation indexes, with an OR of 98.85%. In comparison, the Resnet101 classification network does not show good feature expression ability for the small target objects in the assembly behavior images due to the continuous convolution and pooling operations. Hence, the recognition ability of the model is the lowest. SE-Resnet101 has a similar structure to Resnet101, and the difference is that the former adds a channel attention mechanism to the convolution block, which improves the recognition performance to a certain extent. Besides, ML-GCN uses the GCN network to map the potential dependencies between the labels to the image feature classifier so as to improve the recognition ability of the model. Thus, the recognition performance of ML-GCN is better than the Resnet network, with CF1, OF1, CP, CR, OP, and OR of 94.67%, 93.16%, 95.07%, 94.27%, 93.07%, and 93.26%, respectively. Compared with ML-GCN that uses static graph convolution to model the relationship between labels, ADD-GCN uses dynamic graph convolution, and it can automatically model the adjacency matrix in graph convolution according to image features. The OF1 and CF1 of ADD-GCN reach 97.09% and 96.75%, respectively. Considering the characteristics of small target objects and complex backgrounds in assembly behavior images, the AM-GCN proposed in this paper adds channel and space attention mechanism and multi-scale feature fusion to the Resnet101 network architecture, which alleviates the above problems to a certain extent. Besides, the dynamic graph convolution module in the ADD-GCN network architecture is adopted to adaptively capture the category relationship between the target objects in the image, thereby further improving the recognition ability of the proposed network. Except for a slightly lower value of CR, the OF1, CP, OP, and OR of AM-GCN is respectively 1.53%, 0.75%, 1.72%, and 1.34% higher than that of ADD-GCN.

This paper also compares AM-GCN with other image recognition models for recognizing each type of assembly action in terms of the AP index. The comparison results are listed in Table 1. In Table 1, A–O respectively represent 15 types of workshop behaviors, including wearing work clothes, wearing work hats, cleaning, stacking materials, lifting materials, inspection records, filing workpieces, picking up parts, hammering heavy objects, using communication equipment, violent handling, smoking, wiping sweat, resting, and drinking water.

**Table 1:** Comparison of performance (%) between AM-GCN and other recognition methods.

	A	B	C	D	E	F	G	H	I	J	K	L	M	N		O
ResNet101	94.2	96.4	82.1	64.2	70.0	92.4	91.7	84.2	93.7	78.6	84.0	61.4	83.6	96.7	61.2	
SE-ResNet101	97.8	95.6	89.4	74.8	77.0	96.4	96.4	89.6	98.0	89.1	89.5	77.8	89.2	97.6	72.2	
ML-GCN	98.0	96.2	92.4	76.5	**87.7**	97.3	97.5	93.8	98.5	89.3	91.9	81.6	92.0	**98.6**	75.2	
ADD-GCN	96.3	**98.2**	94.9	80.9	85.3	97.7	98.2	95.9	**98.6**	92.5	93.4	84.6	95.1	99.5	78.8	
AM-GCN	**99.0**	98.1	**95.4**	**81.1**	85.4	**97.8**	**98.5**	**96.7**	97.6	**94.7**	**93.8**	**84.8**	**96.0**	98.2	**79.6**	

As shown in Table 1, the proposed AM-GCN network model achieves the best accuracy in most cases. Compared with the ADD-GCN network, the accuracy of the network model in this paper is improved by 2.2% on recognizing the behavior of using communication equipment. The recognition accuracy of the smoking behavior is relatively low at 79.6%. The reason may be that cigarettes occupy a small proportion in the entire image, which makes recognition difficult. Besides, compared with other models, the AM-GCN network model has improved the recognition of most assembly behaviors to varying degrees.

**Figure 9:**
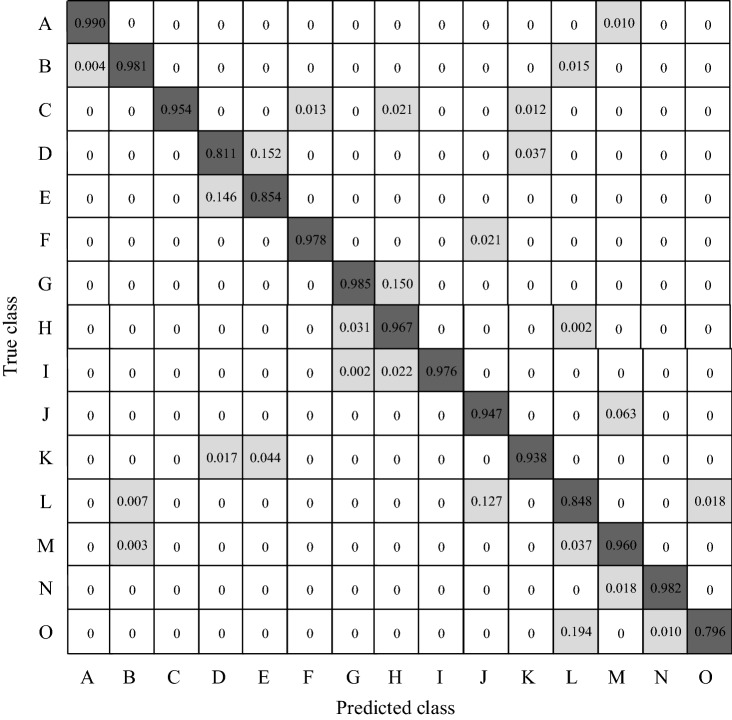
Confusion matrix of assembly behaviors.

This paper draws the confusion matrix according to the accuracy of the proposed network model on the test set (Fig. 9). As shown in Fig. 9, the proposed AM-GCN network model achieves good performance in most assembly behavior recognition cases. The accuracy of recognizing the behaviors of wearing tooling and filing workpieces can reach 99.0% and 98.5%. The recognition accuracy of some behaviors is low, e.g., the recognition of smoking and drinking behaviors only obtain an accuracy of 79.6% and 84.8%, respectively. It may be because the human posture motions involved in these two types of movements are arm movements with a relatively large similarity. Also, the cigarettes and water cups occupy a small proportion in the image, which makes it difficult to recognize.

To test the effectiveness of the proposed AM-GCN, extensive experiments are conducted using benchmarking Microsoft COCO dataset^37^. The Microsoft COCO dataset is usually used for object segmentation and detection. Recently it was also used in image recognition research. It consists of a training set with 82,081 images and a validation set with 40,137 images. The dataset covers 80 common object categories, which makes MS-COCO dataset more challenging. We compared AM-GCN network model with other methods on the validation set. The evaluation metrics include CR, CP, OP, OR, OF1 and CF1. As shown in Table 2, the AM-GCN network model proposed in this paper also shows good performance on Microsoft COCO dataset. Compared with the ResNet-101 network model, all evaluation indicators have been significantly improved, and the OP indicator has increased by 8.8%. Compared with ML-GCN, the OR index improved by 1.9%. Compared with the ADD-GCN model, although the OP index reaches 85.5%, but other indicators are lower than those of ADD-GCN.

**Table 2:** Comparison of performance (%) on COCO dataset.

	CP	CR	CF1	OP	OR	OF1
ResNet101^33^	76.1	68.4	70.0	76.7	70.5	73.5
ML-GCN^29^	85.1	72.0	78.0	85.8	75.4	80.3
ADD-GCN^30^	84.7	75.9	80.1	84.9	79.4	82.0
Ours	84.6	72.6	78.1	85.5	77.3	81.2

## Conclusion and future work

Production process monitoring is crucial to the production efficiency and production safety of the workshop. This paper proposes a dynamic graph convolutional network for assembly behavior recognition based on multi-scale feature fusion and attention mechanism. This method introduces a multi-scale receptive field module to the Resnet101 backbone network, which alleviates the problem of global information loss caused by the deepening of the network layer. Meanwhile, the channel and spatial attention mechanism is introduced into the residual module, the channel and spatial dimensions are weighted, and the output of the residual learning block is changed, so that the attention of the convolutional neural network can be better focused on the target area thereby enhancing the network’s extraction of key information in the feature map and improving the category discrimination ability of the model. Besides, the dynamic graph convolutional network takes the features extracted by the attention module as input, and it adaptively captures the category relationship between different assembly behaviors to recognize the behaviors of workshop personnel. The established model is tested on the ABDS data set and compared with four deep learning-based behavior models. The results show that the network model proposed in this paper achieves good performance for recognizing the production behaviors of workshop personnel.

Certainly, the method proposed in this paper has certain shortcomings. The future work will focus on the following two aspects.

(1) Currently, the data set collected in this paper contains 15 types of assembly behaviors. More assembly behaviors need to be added to realize the deployment of the workshop. Also, the problem of occlusion should be considered when collecting assembly behavior data sets.

(2) The network model designed in this paper has high recognition efficiency and can achieve real-time assembly behavior recognition. However, it has a demand for hardware, and the cost to deploy it in the workshop is high. In the future study, the network model will be optimized to reduce its complexity.
